# The limited effect of information on Israeli pregnant women at advanced maternal age who decide to undergo amniocentesis

**DOI:** 10.1186/s13584-015-0019-6

**Published:** 2015-08-17

**Authors:** Julia Grinshpun-Cohen, Talya Miron-Shatz, Michal Berkenstet, Elon Pras

**Affiliations:** The Danek Gertner Institute of Human Genetics, Sheba Medical Center, Tel Hashomer, Israel; Sackler Faculty of Medicine, Tel Aviv University, Tel Aviv, Israel; Center for Medical Decision Making, Ono Academic College, Kiryat Ono, Israel; Center for Medicine in the Public Interest, New York, NY USA

**Keywords:** Advanced maternal age, Informed choice, Amniocentesis, Down syndrome risk

## Abstract

**Background:**

A primary goal of amniocentesis is the detection of trisomy 21 (Down syndrome- DS) in the fetus. This procedure involves a small risk of miscarriage. As the risk of DS increases with maternal age, screening tests (maternal serum triple test and others) and age are used to generate a risk assessment, and amniocentesis is offered to women with high risk. In Israel, amniocentesis is government funded for women of advanced maternal age (AMA, i.e., ≥35 years), even if their risk assessment is low.

The purpose of this study was to explore the reasons AMA women undergo amniocentesis, their knowledge about risk estimates, and to evaluate whether their decision is informed.

**Methods:**

Shortly after undergoing amniocentesis, 42 consecutive women without a medical indication for amniocentesis other than age, completed a questionnaire that assessed their knowledge and opinions regarding screening tests, pregnancy termination, amniocentesis risks and the factors that affected their decision.

**Results:**

Women rarely deliberated before undergoing amniocentesis. One third of those who had the screening test did not wait for the results before undergoing amniocentesis. Only one third of those who received the screening results remembered their risk estimation before going ahead with amniocentesis. Almost half (41 %) cited “age” as their main reason for undergoing amniocentesis, though only 44 % of these women could recall their age related DS risk. Sixty percent estimated their DS risk as low or very low but still had amniocentesis. Most participants (74 %) stated that they would consider termination of the pregnancy if the fetus was diagnosed with an intellectual deficit.

**Conclusions:**

These results cast doubt on whether AMA women’s decision to undergo amniocentesis is based on risk estimates, as women seem to disregard risk estimates, and sometimes not even wait for them when making the decision. The policy of funding amniocentesis solely on the basis of age may have led to the conception that being over 35 alone is sufficient reason to undergo amniocentesis. This finding should inform policy makers, as it raises questions about the link between public funding and the choices of individual women, and has implications for healthcare expenditures.

## Background

Medicine increasingly emphasizes patient participation and informed choice in decision making, which sometimes lead to financial savings [1]. O’Connor and O’Brien-Pallas state that “An effective decision is one that is informed, consistent with the decision maker’s values and behaviorally implemented” [2]. This study set out to examine the degree to which the decision of Israeli women who are AMA (advanced maternal age, i.e., ≥35 year old) to undergo amniocentesis is informed by risk estimates and the factors that affect this decision.

Amniocentesis is an invasive procedure performed during pregnancy to determine (among other things) whether the fetus has Down syndrome (Trisomy 21- DS). The risk of fetal DS increases with increased maternal age, from 1:1600 at age 20 to 1:30 at age 45, with a risk of approximately 1:350 at age 35 and 1:100 at age 40 [3, 4]. Screening for DS in maternal serum – such as the triple screen performed in the second trimester that includes measurement of AFP (Alfa Fetoprotein), HCG (Human Chorionic Gonadotropin) and UE3 (Unconjugated Estriol) – facilitates better risk estimates and identification of subgroups of women considered to be at higher risk for whom amniocentesis is indicated. Conversely, DS screening also facilitates identification of those at low risk for DS for whom amniocentesis is not recommended [5]. Amniocentesis is associated with an increased risk of miscarriage. The exact magnitude of this additional risk is equivocal and is estimated to be in the range of 1:100–1:1600 [6–8]. While various risk estimates are used by physicians and counselors (in Sheba Medical Center 1:1000 is the risk of miscarriage commonly conveyed during counseling), the Israeli Medical Association consent form used by all Israeli medical facilities gives the risk of miscarriage as 1:200. Thus, the decision to perform the procedure can also lead to negative consequences.

Since 1980, amniocentesis has been funded by the Israeli Ministry of Health for women who are older than 37 years at the onset of pregnancy regardless of other indications [9], and since 1993, this funding has been expanded to all women ages 35 and up [10, 11]. Additionally, amniocentesis is funded by the Ministry of Health for women younger than 35, whenever there are abnormal ultrasound findings or relevant family history, and by the health insurance organizations when there is a high risk based on screening tests [12, 13].

During the period of 2002–2006, almost 47,500 women in Israel had amniocentesis for the sole indication of AMA. In 2006, 11,131 amniocentesis procedures were performed in Israel due to AMA, while only 2355 procedures were performed for all other medical indications, such as ultrasound findings or high risk for a genetic disease (not including high DS risk) [14]. Compared with other western countries, Israeli women tend to perform more tests during pregnancy including genetic carrier screening, ultrasound scans and biochemical screening for DS [15]. Our previous work has shown that, in a small sample of educated, mostly secular AMA women, the vast majority (87 %) had amniocentesis; many of them performed the test before receiving their DS screening results [16]. Based on the latest survey of the Israeli Ministry of Health, among Jewish women under age 35, 62 % had triple test screening and 11 % had amniocentesis [17]. However, among Jewish AMA women, 47 % had amniocentesis (and 64 % had triple test screening). Among AMA women that did not have amniocentesis, 83 % said that they were referred for it [17]. This survey included religious women who are known to perform fewer tests,

Studies from the Netherlands, Australia and Canada showed that only a fraction of women reached an informed decision regarding screening for DS [18–20]. These studies suggest that in order to promote a higher rate of informed decisions, counselees need professional help in obtaining the necessary information and exploring their options. Furthermore, the studies propose that risk perception is complicated and that risk estimates are often not directly integrated into the decision making process. In a study conducted before screening for DS was available, French et al. found no relationship between knowledge and the decision to undergo amniocentesis in a sample of highly educated women [21]. Even when DS screening was offered, it was noted that pregnant AMA women’s decision regarding amniocentesis may not always correlate clinically with DS screening results [22], though some researchers have found that screening for DS decreased amniocentesis uptake among AMA women [23, 24].

If the decision to undergo amniocentesis is not fully informed by objective risk estimates, and there is no clear association between DS screening results and women’s decision to undergo amniocentesis, what drives the decision? Previous work has suggested several factors that predict test uptake: positive attitudes toward prenatal diagnostic tests, perception of prenatal diagnostic tests as reliable, and requests for more scientific information [25]. A study conducted in Israel found that use of amniocentesis by low risk women was associated with older age and receipt of more information about the test [26]. Others have shown that negative perceptions of having a child with DS had a more significant influence on decisions than the numeric probability of having such a child, as indicated by screening results [27]. In a previous study, we found that *a priori* opinions regarding the relationship between age and amniocentesis risk, as well as termination tolerance, affected the decision more than test results obtained during pregnancy [28]. Additionally, Bryant et al. have shown that attitudes toward having a child with DS were significantly associated with uptake of screening, as well as with intention to undergo amniocentesis and willingness to terminate an affected pregnancy [29, 30]. In sum, the relationship between screening tests and amniocentesis uptake is complex, and the decision to undergo amniocentesis is often not based on medical or statistical information, thereby raising the question of what drives the decision and how informed it is.

The objectives of the current study were to outline the factors that affect Israeli AMA women’s decision process regarding amniocentesis and to explore whether the decision is informed by knowledge, is carried out by considering the risks, and is aligned with a willingness to terminate a pregnancy if a problem is detected with the fetus. Degree of deliberation was also used as an indirect measure of informed choice. It has been previously shown that deliberation can be used to measure decision quality [31], and interventions that help people deliberate, promote a better decision process [32]. In addition to a measure of informed choice, degree of deliberation was also examined in terms of its association with decision-relevant factors. Based on our previous work [16], we expected women to have a low level of knowledge regarding the many tests they had during pregnancy. Since knowledge is a central component of informed choice, their decision was predicted not to be fully informed. Previous studies did not extensively address variables like objective risk perception and termination tolerance. These were used here to explore whether the decision is concordant with personal (subjective) perceptions and beliefs, even if it is not fully informed.

## Methods

### Procedure

The study was conducted at the Sheba Medical Center after being approved by the Sheba IRB. For several days, all AMA women who had amniocentesis were approached after the procedure and asked to participate. Those who agreed signed a consent form and completed a 30-question questionnaire that took approximately 15 min. Initially, fifty participants were recruited of the 57 who were approached; thus participation rate was 87 %. Seven participants were later excluded due to abnormal screening results or ultrasound findings, and one was excluded due to twin pregnancy. Thus, 42 AMA participants who decided to undergo amniocentesis without a medical indication were interviewed.

### Participants

All 42 participants were Hebrew speakers. The average age was 37.8 years (range 35–42, SD = 1.94). Almost all participants (*n* = 38, 90 %) had previous children, and eight (19 %) had undergone fertility treatments for the current pregnancy. Participants were highly educated: 23 (55 %) had a college degree and 17 of those (40 %), had also completed post-graduate degrees (MS, MD or PhD). Most participants (*n* = 34, 81 %) described themselves as secular, six (14 %) as traditional and two (5 %) as religious.

### Measures

1. Knowledge score - a 0-4 score that captured recall of the age-related and amniocentesis-related risk estimates from the first trimester screening and second trimester screening.
2. Age-risk perception - the subjective age-related DS risk, as scored on a 5 point scale (very low-1 to very high -5).
3. Amniocentesis risk perception - defined depending upon whether the woman perceived the risk associated with amniocentesis as (1) low, (2) medium or (3) high.
4. Deliberation level - whether the woman reported having much (score of 2), some (score of 1) or no (score of 0) deliberation regarding her decision to undergo amniocentesis.
5. Pregnancy termination tolerance - was a 0-5 score defined as a cumulative number of “yes” answers (coded as “1”) to the question of termination of pregnancy if the fetus were diagnosed with:
  - A surgically correctable heart defect
  - A condition associated with an intellectual deficit
  - A condition associated with infertility
  - A condition associated with severe disabilities and death within the first year of life
  - A disease that starts after age 40 and progresses to motor disability
6. Personal reasons for amniocentesis –an open-ended question: “What were the determining factors that made you decide to have amniocentesis?” Answers were examined for common themes and grouped into several categories. If more than one factor was mentioned in a single response, they were each counted separately.

### Statistics

Statistical analysis was performed using SPSS v19 and included: bivariate Pearson correlations among level of deliberation, age-risk perception, termination tolerance and amniocentesis risk perception. Multivariate logistic regression was also performed for these variables, treating level of deliberation as the dependent variable. Statistical significance was determined at p ≤ 0.05.

## Results

Questionnaires were analyzed while exploring several topics to assess different aspects of the decision process and what affects it. In the following sections, we present objective knowledge, subjective perceptions and personal reasons, all of which we expected to influence the decision. These are followed by presentation of decision “quality” with regard to informed choice, deliberation and subjective beliefs.

### Knowledge measures

Only half of the participants (*n* = 21) performed the first trimester screening for DS. A greater number of participants (*n* = 32, 76 %) underwent the second trimester serum screening for DS, but about a third of these participants (*n* = 11) did not wait for the screening results before performing amniocentesis. Four participants (10 %) had not undergone either of the screening tests before having amniocentesis.

Participants’ knowledge was evaluated by asking them to recall several risk estimates relevant to their decision regarding amniocentesis. Specifically, they were asked about the results of the two screening tests (if performed), their age related DS risk and the miscarriage risk associated with amniocentesis. Results for knowledge of risk estimates are presented in Table 1.

**Table 1:** Participants’ knowledge of their risks relevant to the decision regarding amniocentesis

Test	Correctly recalled result	n^a^ (%)
First trimester screen	11	21 (52 %)
Second trimester screen	7	21 (33 %)
Age related risk	15	42 (36 %)
Amniocentesis risk^b^	39	42 (93 %)

^a^For the first and second trimester screens, the percentages are calculated among those who had the test and received results at the time of survey. For age and amniocentesis related risk, the percentages are calculated among all participants

^b^Both 1:1000 and 1:200 estimates of miscarriage risk associated with amniocentesis were accepted as correct

Participants most often recalled the amniocentesis related risk, which was explained to them immediately before the amniocentesis, less than two hours before being interviewed for the study. Almost all participants (93 %) knew this risk. However, recall for the other risk estimates was poorer: e.g., only 36 % of the participants who responded to the question knew their age-related risk. Most participants knew only their amniocentesis-related risk of miscarriage, and only one woman knew all the relevant risk estimates.

### Do subjective views affect the decision to have amniocentesis and deliberation over the decision?

- Subjective risk perception:
  Age-risk perception: Participants’ average age related risk perception was 2.56 (SD = 1.14, on a scale of 1-5). Perceived age-related DS risk was significantly correlated with age (r = 0.52, P < 0.05), so that older women perceived a higher age related risk. However, there was no significant difference in age-risk perception between those who actually knew their age related risk and those who did not. Thus, women appear to have a general notion of increased risk with age even if they lack knowledge of the actual risk level.
  Amniocentesis risk perception: Most participants (*n* = 35, 83 %) recalled their amniocentesis related risk. This was a multiple choice question, and either 1:1000 (the risk cited during counseling) or 1:200 (the risk stated on the consent form) were accepted as correct). Table 2 presents the subjective risk perceptions of these participants who accurately provided risk estimates. Most participants (66 %) considered the risk associated with amniocentesis as low, and only 26 % thought that it was higher than their risk for DS.

**Table 2:** Subjective risk perceptions of women regarding amniocentesis risk

Question:	Replies:	Number of women (percentage of total responders)^a^
“Do you consider the risk associated with amniocentesis as high, medium or low?”	Considers risk high	5 (14 %)
	Considers risk medium	7 (20 %)
	Considers risk low	23 (66 %)
“Which risk do you think is higher, the risk for DS^b^ or the risk of amniocentesis”	Amniocentesis risk higher than DS risk	9 (26 %)
	The two risks are similar	6 (17 %)
	Amniocentesis risk lower than DS risk	15 (43 %)

^a^35 women replied to the first question and 30 to the second

^b^DS is an abbreviation for Down syndrome
- Deliberation regarding amniocentesis and what affects it:
  More than half the participants (*n* = 24, 57 %) did not deliberate over the decision at all, while 18 participants said they deliberated over the decision either somewhat (*n* = 11, 26 %) or very much (*n* = 7, 17 %). These 18 participants had a lower age-risk perception, and there was a significant negative correlation between age-risk perception and degree of deliberation for the entire sample (r = -0.35, *p* < 0.05).
  Termination tolerance showed a significant negative correlation with deliberation (r = -0.14, P < 0.05). Thus, women with a higher tolerance for pregnancy termination spent less time deliberating over amniocentesis. Amniocentesis risk perception (r = 0.17, P = 0.29), age (r = -0.21, P = 0.17), and number of children (r = 0.23, P = 0.14) were not significantly correlated with deliberation.
  When logistic regression was performed for all the above mentioned predictor variables with deliberation (0- none, 1- some or much) as the dependent variable, only age-risk perception was a significant predictor (B = -1.15, P < 0.05).
- Termination tolerance:
  Two participants did not reply to this question. The average termination tolerance score for the remaining 40 participants was 2.5 (SD =1.28) on a scale of 0-5. Thus, the average participant was willing to consider termination in half of the presented scenarios. The most common score was 2 (*n* = 11) followed by 3 (*n* = 10) and 1 (*n* = 9).
  As presented in Table 3, five out of 36 respondents (14 %) answered that they would not terminate the pregnancy for scenario b (a condition associated with an intellectual deficit), which corresponds to DS. Two participants claimed they would not terminate for the most severe scenario (scenario d– a condition associated with severe disabilities and death with the first year of life). One of these two answered “yes” to scenario b.

**Table 3:** Distribution of responses for the various termination tolerance scenarios (*n* = 42 total participants)

Scenario	“Yes” I would consider termination	“No” I would NOT consider termination	No response
A. A surgically correctable heart defect	6 (14 %)	34 (81 %)	2 (5 %)
B. A condition associated with an intellectual deficit	31 (74 %)	5 (12 %)	6 (14 %)
C. A condition associated with infertility	6 (14 %)	33 (79 %)	3 (7 %)
D. A condition associated with severe disabilities and death within the first year of life	35 (84 %)	2 (5 %)	5 (12 %)
E. A disease that starts after age 40 and progresses to motor disability	22 (52 %)	15 (36 %)	5 (12 %)

### Personal reasons for amniocentesis

The participants were asked to state their main reason for undergoing amniocentesis. The most common was “age”, mentioned by 18 participants (43 %). Additional reasons were: “wanting certainty” (*n* = 9, 21 %), “fear of DS” (*n* = 6, 14 %) and “doctor's recommendation” or “medical policy”, mentioned by 5 participants each (12 %).

Among those who stated “age” as their reason, only eight participants (45 %) could recall their age related DS risk. More than half the participants for whom “age” was the reason for choosing amniocentesis, (*n* = 11, 61 %), estimated their DS age related risk as “low” or “very low” regardless of whether they could recall it.

### Is the choice regarding amniocentesis informed by the risk estimates, and is there deliberation?

Knowledge measures of the risk estimates, and deliberation levels were used as indicators of an informed choice.

- Knowledge of the age-related risk, amniocentesis risk and at least one DS risk estimate based on serum screening were deemed sufficient for informed choice regarding undergoing amniocentesis. Only five participants (12 %) knew all of these risks. Fifteen participants (36 %) knew the two most relevant risks (age related DS risk and amniocentesis related pregnancy loss risk). Thus, the majority of women did not have the necessary knowledge to make a decision that is informed by risk estimates with regard to amniocentesis.
- Degree of deliberation was used as a measure of a decision that is thoughtfully achieved. Less than half of the participants deliberated their decision.

There is no significant association between knowledge scores and amount of deliberation before amniocentesis (r = -0.095, P = 0.549). Thus, deliberation was independent from knowledge.

### Is the decision concordant with subjective beliefs?

The hypothesis was that women who perceived their DS risk to be higher than the amniocentesis risk (regardless of whether this perception was true) would make a decision concordant with their risk perception. In the entire sample, 18 participants (44 %) perceived a higher risk of DS, 10 (24 %) perceived a higher risk of amniocentesis and 7 (16 %) perceived the risks to be similar (the remaining seven participants did not answer the question). Thus, most participants (60 %) had a perception of risks that supported their decision to undergo amniocentesis. However, 25 participants (60 %) claimed that the relative risk of DS as compared with the miscarriage risk associated with amniocentesis had no effect on their decision.

## Discussion

This study explored whether Israeli AMA women who elect to undergo amniocentesis without a medical indication are making an informed choice, i.e., a decision that is deliberated, and based on relevant information, both subjective and objective. The results show that, for the most part, these women disregard the actual risk estimates and other medical information when making the decision to undergo amniocentesis. Most did not deliberate before making this decision and claimed that the risk estimates were irrelevant for their decision. These results cast doubt on whether these women’s decision to undergo amniocentesis was indeed fully informed. However, as many other reasons affect this decision we did not attempt to explore its subjective “rationality” or validity for these women.

The most common reason participants gave for undergoing amniocentesis was “age” (given by 43 %) but more than half could not recall their age related DS risk, and many considered this risk low and thus not representing an objective age-related reason. It may be that the longstanding policy, which provides funding for amniocentesis based on age alone, has led women and physicians to the perception that the government finds it necessary for women over 35 to undergo amniocentesis. According to a recent survey, over 60 % of Israeli obstetricians would recommend amniocentesis to AMA women with normal screening results [33]. Thus, it may be that physicians’ recommendations also follow the age based funding policy, so that women who followed their physicians’ recommendation were in fact being guided by the government policy. This suggestion is somewhat supported by the finding that five participants (12 %) specifically cited “public policy” as their reason for amniocentesis, and another five (12 %) cited “physician recommendation” as their reason for amniocentesis.

In a previous study (16) we interviewed AMA and young women (without a medical indication for amniocentesis) and found that the majority of AMA women had amniocentesis while citing “age” as their main reason for the test. These women also exhibited low levels of knowledge of the relevant risk estimates. Younger women however, rarely chose to have amniocentesis and had better knowledge of the relevant risks. These finding support our suggestion that there is a widespread perception that age ≥35 is by itself a reason to have amniocentesis regardless of other factors.

Some elements of an informed decision were evident: many participants believed that their DS risk was higher than the risk of amniocentesis, thus constituting subjective justification for having the procedure. In addition, most participants stated that they would consider termination of a pregnancy affected with intellectual disability, consistent with the notion that the purpose of amniocentesis is to diagnose fetuses with DS and provide an option to terminate these pregnancies. However, concordance of a decision with subjective views and beliefs is not equivalent to an informed decision, such that is based on risk estimates, as the belief that the risk of DS is higher than the risk of amniocentesis was not based on the actual risk levels.

The results are in general agreement with the findings of Vergani et al. [34] who reported that the key determinant of the choice to have amniocentesis among AMA women (whether they had the procedure or not) is the *a priori* opinion of the woman towards the procedure. Most participants seemed to have had a predetermined decision, and deliberated little about it during pregnancy, irrespective of their actual risk estimates.

Notably, women may have other reasons for amniocentesis, and the magnitude of DS risk is only one of their considerations. This small-scale study did not explore participants’ views on having a child with disability and the relative burden of raising such a child in comparison to losing a pregnancy due to miscarriage. It is possible that some women consider the possibility of a DS child as unacceptable and are not willing to take any risk of it happening, thus performing amniocentesis regardless of their risk level. This issue warrants further exploration in a larger study.

The findings show that while most participants had tests that would provide screening information regarding DS risk, a considerable portion of them did not wait for the test results before going ahead with amniocentesis. This suggests that women do not consider screening tests as necessary to inform their decision regarding amniocentesis, and some women decide not to undergo the screening tests at all, as they have already decided to undergo amniocentesis, mostly due to “age”, but also due to a desire for certainty, fear of DS, and physician recommendations. Given the relative lack of deliberation and consideration of risks associated with the procedure, policy makers should consider whether sweeping funding for amniocentesis for AMA women sends the message that the procedure is warranted and without risk. In this context, promoting shared decision making (SDM) in the Israeli health system as stated by Miron-Shatz et al. [35] may be important. Promoting SDM has also been previously suggested as a way to reduce healthcare costs [1].

The present study has several limitations. The sample was relatively small, featuring 42 women from one medical center, who were not fully representative of the general Israeli population. However, this pilot study reasonably represents a large segment of secular, educated, Israeli women who utilize many prenatal tests, as was found by the Ministry of Health survey [17]. The Ministry of Health survey shows that while there is no significant difference in utilization of other prenatal tests between AMA and younger women, the difference regarding amniocentesis is striking. However, the extensive survey conducted by the Ministry of Health did not address women’s reasons to pursue various tests, nor did that survey evaluate the results of these tests so that the actual risk levels of the women that did or did not have amniocentesis are unknown. Due to the small size of the sample in the present study, we cannot assume generalizability to the entire Israeli population, so it may be valuable to address this in a future nationwide survey. Another limitation is that the present study only examined women who had amniocentesis. Future work should also explore the factors influencing decision-making in women who did not have the procedure, thereby allowing a comparison between the two groups.

New technologies, such as noninvasive testing for fetal trisomy and microarray-based techniques, have recently been introduced into the prenatal testing field. Many technologies are offered directly to consumers under the premise that the consumers can make informed decisions about them. However, our findings cast doubt on whether women take into account all risk estimates available to them in such circumstances. The integration of these technologies into existing practices should be designed to address these issues by both the medical community and policy makers.

## Conclusions

The results of this study cast doubt on whether the decision to undergo amniocentesis after age 35 is fully informed by risk estimates among women that had the procedure. The longstanding policy which provides funding for amniocentesis based on age alone may have led to the conception that being over 35 is, in and of itself, a sufficient reason to undergo amniocentesis. Thus most women do not deliberate before having the procedure, and some go through the motions of DS screening, but do not take the results into account when deciding to undergo amniocentesis.

This information is significant for policymakers as it emphasizes the link between public funding and the choices of individual women, and thus has implications for allocation of healthcare expenditures.
